# Thrombocytopenia and disseminated histoplasmosis in immunocompetent adults

**DOI:** 10.1002/ccr3.1182

**Published:** 2017-10-18

**Authors:** Issa Kutkut, Laura Vater, Mitchell Goldman, Magdalena Czader, Jessica Swenberg, Zachary Fulkerson, Rakesh Mehta

**Affiliations:** ^1^ Department of Internal Medicine Indiana University School of Medicine Indianapolis Indiana; ^2^ Division of Infectious Diseases Department of Internal Medicine Indiana University School of Medicine Indianapolis Indiana; ^3^ Department of Pathology and Laboratory Medicine Indiana University School of Medicine Indianapolis Indiana; ^4^ Indiana University Health Physicians Family Medicine Zionsville Indiana; ^5^ Division of Hematology‐Oncology Department of Internal Medicine Indiana University School of Medicine Indianapolis Indiana

**Keywords:** Fungal, histoplasmosis, mycoses, pancytopenia, thrombocytopenia

## Abstract

Disseminated histoplasmosis among immunocompetent patients is rare, but may be associated with clinically significant refractory thrombocytopenia. Platelet counts often return to normal levels following antifungal therapy. Therefore, the most important management of this refractory thrombocytopenia is the recognition and treatment of histoplasmosis infection.

## Case Presentation and Clinical Reasoning

A 47‐year‐old man presented with a 6‐week history of episodic fevers associated with cramping abdominal pain. Fevers occurred three times daily, were accompanied by severe abdominal pain across the upper right and left quadrants, and were followed by drenching sweats and chills. The patient also complained of nausea, dry cough, and an 18‐kg weight loss during this time. Other than cholecystectomy and knee ligament surgeries, the patient had been previously healthy with no recent or recurrent infections or known hematologic abnormalities. He was adopted and had no information about the health of his family members. He lived in Indiana and worked in an office building near heavy construction. Four months prior, he traveled to the Bolivian jungle for a fishing trip where he consumed meat and was exposed to insects, but did not swim or drink contaminated water.

The patient's presentation with fevers, night sweats, and weight loss over a 6‐week period raises concern for a chronic infection, malignant process, or rheumatologic disease. His recent history of international travel makes virulent tropical disease an important diagnostic consideration in this previously healthy patient. Exposure to insects raises suspicion for mosquito‐borne or sandfly related diseases such as malaria, dengue fever, chikungunya, or yellow fever as well as visceral leishmaniasis. Consumption of undercooked meat may cause trichinosis or toxoplasmosis, conditions that may present with fever and abdominal pain.

**Figure 1:**
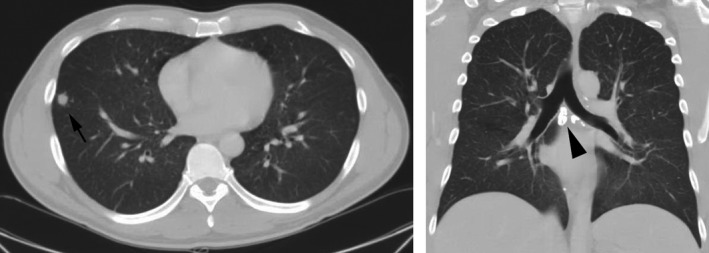
Chest computerized tomography (CT) scan with contrast showing numerous bilateral pulmonary nodules (arrow: 1‐cm nodule) and calcified mediastinal and hilar lymph nodes (arrowhead: mediastinal lymph nodes).

**Figure 2:**
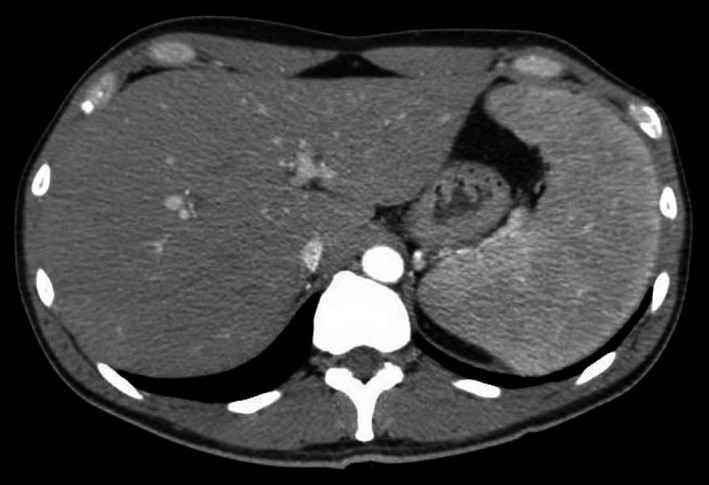
Abdominal and pelvic CT scan with contrast revealing moderate splenomegaly and trace ascites.

Prior to the present admission, the patient had two brief hospitalizations at another facility and underwent an extensive infectious disease evaluation. Diagnostic tests were performed for HIV infection, malaria, dengue fever, chikungunya, hepatitis B and C, cytomegalovirus, and Epstein–Barr virus, all of which were negative. Laboratory results revealed elevated liver transaminases, which peaked 1 month before admission at our hospital (aspartate aminotransferase [AST] 302 units/L, alanine aminotransferase [ALT] 517 units/L). A right upper quadrant ultrasound was unrevealing, and head computerized tomography (CT) scan and cerebrospinal fluid analyses were negative. Chest CT scan with contrast revealed numerous bilateral pulmonary nodules and calcified mediastinal and hilar lymph nodes (Fig. 1). An abdominal and pelvis CT scan with contrast showed moderate splenomegaly and trace ascites (Fig. 2). Given his imaging findings, elevated liver transaminases, and history of abdominal pain, he was referred to the hepatology service at our hospital for further evaluation.

The patient's unrevealing initial infectious disease evaluation in combination with bilateral pulmonary nodules and calcified lymph nodes make sarcoidosis and granulomatous infections such as histoplasmosis, coccidioidomycosis, and blastomycosis as well as tuberculosis important considerations in this patient. Sarcoidosis may cause granulomatous infiltration of the spleen and liver, leading to elevated liver transaminases. Endemic fungal infections also commonly involve the liver and spleen. Tuberculosis, mycobacterium avium‐intracellulare infection, visceral leishmaniasis, and secondary syphilis may also cause granulomatous hepatitis.

On admission, the patient was afebrile and in mild distress, with no appreciable lymphadenopathy, petechiae, or ecchymoses. Initial laboratory serum results were notable for a WBC count of 3.2 k/cumm, with 65% neutrophils, 18% lymphocytes, and 13% monocytes, a hemoglobin level of 11.7 g/dL, and a platelet count of 4 k/cumm. Prothrombin and partial thromboplastin times were normal. The peripheral blood smear revealed severe thrombocytopenia with normal platelet morphology and toxic granulation. Platelet‐associated antibody screening revealed 70% reactivity. Results also showed slightly elevated lactate dehydrogenase (305 units/L, reference range 140–271 units/L) and elevated ferritin (1396 ng/mL), lysozyme (14.8 μg/mL), and angiotensin‐converting enzyme (152 units/L). Liver transaminases were slightly elevated (AST 52 units/L, ALT 58 units/L), and alkaline phosphatase was also elevated (202 units/L).

The patient's laboratory results reveal pancytopenia with severe thrombocytopenia, as well as toxic granulation on peripheral blood smear, suggestive of infectious or inflammatory process. The patient's elevated level of angiotensin‐converting enzyme makes sarcoidosis an important diagnostic consideration, although elevated levels may also be found in tuberculosis or endemic mycoses. Markedly elevated ferritin in the setting of normal iron levels also suggests an acute or chronic inflammatory process or malignancy.

**Figure 3:**
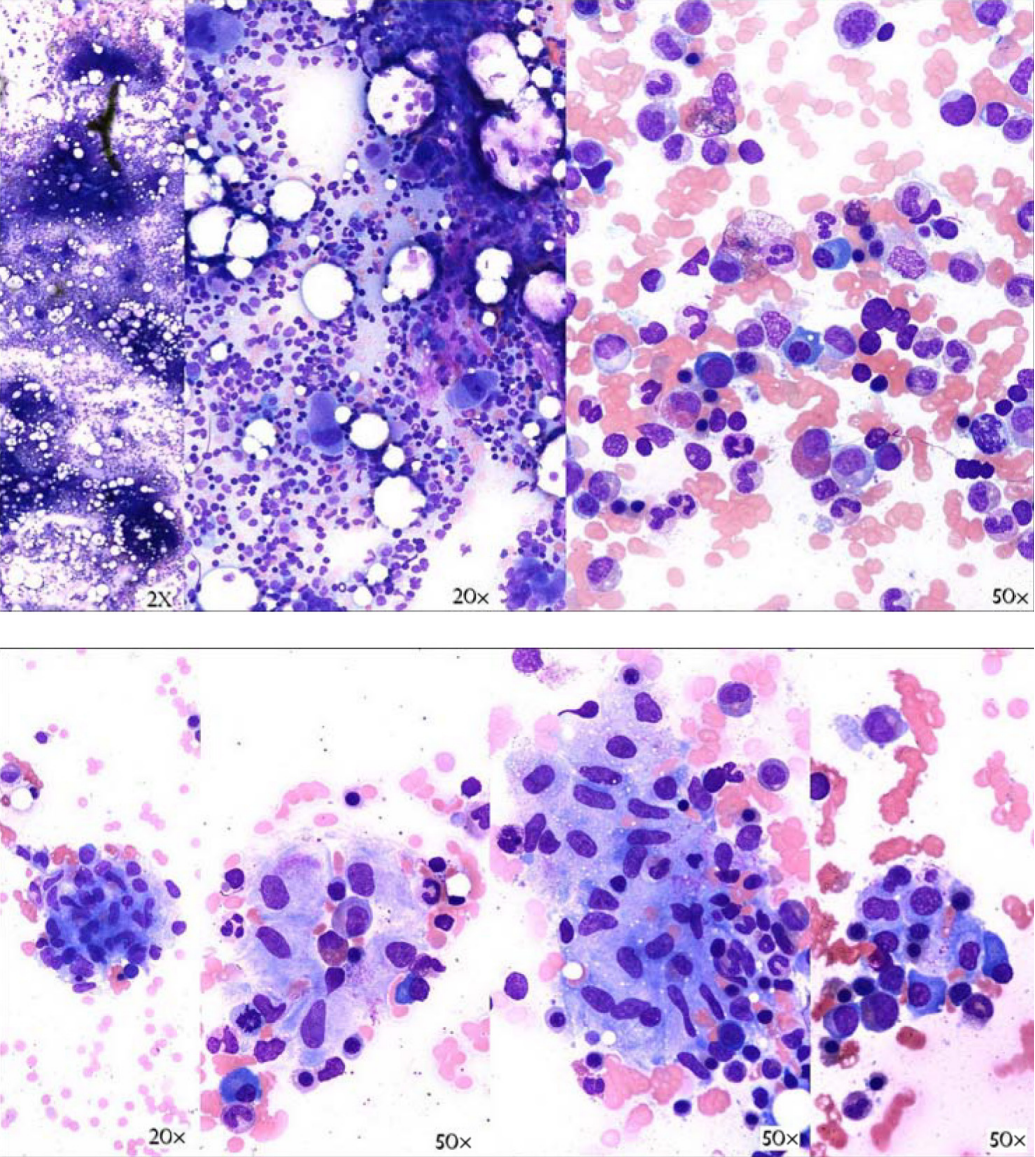
Bone marrow aspirate with clusters of epithelioid histiocytes and increased megakaryocytes. Mildly increased megakaryopoiesis is best shown in the middle image (20×).

**Figure 4:**
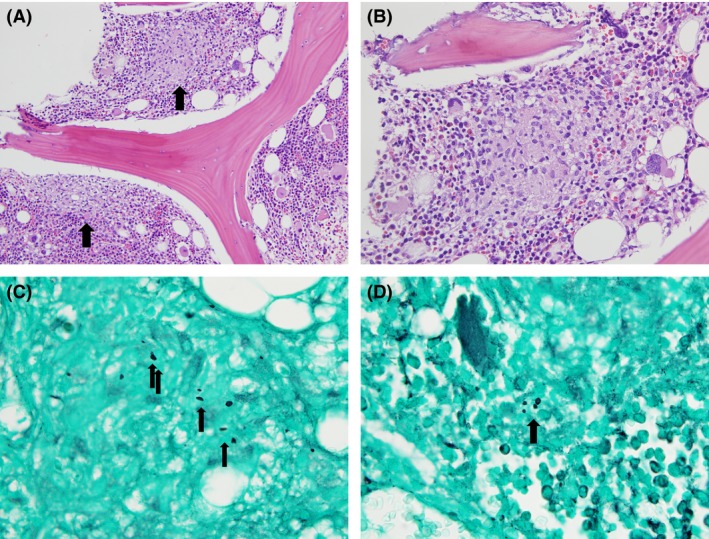
(A) Bone marrow biopsy showing hypercellular bone marrow with scattered granulomata (arrows) and maturing trilineage hematopoiesis (H&E stain, 200×). (B) Bone marrow biopsy with granuloma including epithelioid histiocytes, lymphocytes, and plasma cells (H&E stain, 500×). (C) + (D) Scattered fungal forms (arrows) were seen in granulomatous foci (GMS stain, 1000×).

Fungal and mycobacterial studies were sent, and bone marrow aspiration and biopsy were performed. The *Histoplasma capsulatum* urine antigen, serum antigen, and serum antibody test results were all positive. Bone marrow aspirate showed clusters of epithelioid histiocytes and increased megakaryopoiesis with mild dysmegakaryopoiesis (Fig. 3). Biopsy showed hypercellular bone marrow with scattered granulomata including epithelioid histiocytes, lymphocytes, and plasma cells (Fig. 4A and B). Acid‐fast bacillus and Grocott's methenamine silver (GMS) stains were performed. The GMS stain showed yeast forms (Fig. 4B and C), and the bone marrow culture was positive for *H. capsulatum*. The bone marrow aspirate flow cytometry showed no evidence of malignancy. The hematology team was consulted, and workup for common variable immunodeficiency with quantitative immunoglobulin levels was negative.

The dimorphic fungus *H. capsulatum* is found in soil around the world, most commonly in South America and the Ohio and Mississippi river valleys of the United States 1. While those living in endemic areas are exposed to *H. capsulatum* at high rates, symptomatic infection is uncommon. Clinical manifestations occur in <1% of exposed persons and include acute pulmonary disease, chronic cavitary disease, and disseminated disease 2. Disseminated histoplasmosis is the rarest form of infection and typically presents with fever, malaise, weight loss, and lymphadenopathy among immunosuppressed persons 2. While uncommon, disseminated disease does occur in adults without any recognized immunologic deficits and has been associated with a profound thrombocytopenia 3, 4.

**Figure 5:**
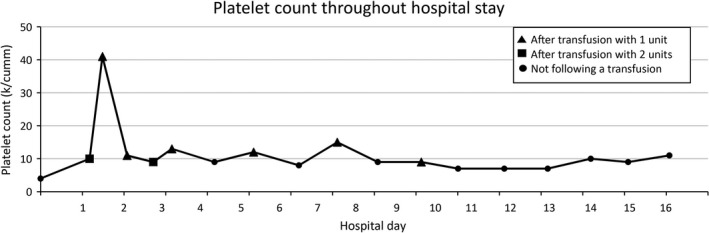
Trend of platelet count throughout hospital stay.

**Figure 6:**
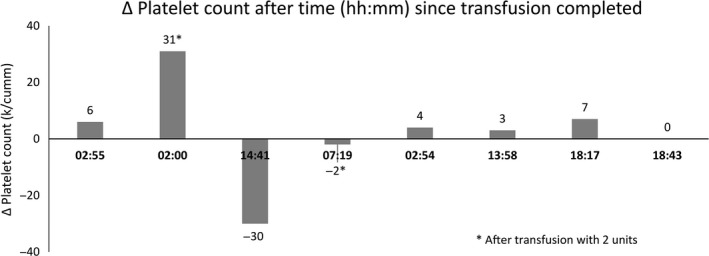
Change in platelet count in relation to time after transfusion.

Intravenous liposomal amphotericin 3 mg/kg/day was started on day 2 of admission for 14 days. Itraconazole 200 mg twice daily was started on day 4 with instructions to continue treatment for 1 year. The patient was no longer febrile by day 4 and noted improvement in strength and energy by day 6. While the white blood cell count and hemoglobin level gradually increased starting on day 7, the platelet count did not respond to several platelet transfusions (Figs. 5 and 6). The patient initially received two doses of 1 g/kg IV immunoglobulin (IVIG) over 2 days with no improvement. Prior to discharge, the platelet count finally stabilized around 10 k/cumm (Fig. 5). Platelet count was monitored with serial checks at the hematology clinic during the course of antifungal treatment.

Corticosteroids were not administered as the patient did not respond to IVIG, he was not actively bleeding, and he had a severe disseminated histoplasmosis infection. It was also decided to hold further platelet transfusions unless the platelet count dropped below 5 k/cumm or in the case of bleeding, neither of which occurred.

One month following discharge, the *H. capsulatum* urine antigen remained positive, but the patient reported considerable clinical improvement with no further febrile episodes or abdominal pain. His platelet count was 39 k/cumm at the time. Two months after discharge, laboratory results showed a normalization of the platelet count (221 k/cumm) and liver function tests. The *H. capsulatum* urine antigen test was negative 5 months following hospital discharge. The patient completed 1 year of itraconazole therapy, and he experienced no treatment side effects. The *H. capsulatum* urine antigen remained negative 6 weeks following treatment cessation. The patient returned to his baseline of functioning and has remained healthy to date.

The patient's thrombocytopenia was thought to be secondary to disseminated histoplasmosis, and he was discharged from the hematology clinic. Liposomal amphotericin B is recommended for severe cases of disseminated histoplasmosis 1. The lipid formulations require 3–5 mg/kg/day, and the deoxycholate formulations require a dose of 0.7–1.0 mg/kg/day. As in the case of our patient, most patients respond quickly to amphotericin B treatment and can be transitioned to itraconazole therapy. The ideal duration of itraconazole treatment is currently not known, but therapy for at least 1 year may reduce the risk of relapse infection.

## Discussion

We report a case of disseminated histoplasmosis in a presumably immunocompetent male complicated by severe thrombocytopenia. While disseminated disease typically occurs in immunosuppressed patients, we found no evidence of an immune deficiency and the patient has remained healthy to date. Prior to disease onset, the patient had been working in an office building adjacent to a construction site. Heavy aerosol exposure to *H. capsulatum* can produce an influenza‐like syndrome of acute pulmonary histoplasmosis 5. Although the patient had numerous bilateral pulmonary nodules and calcified mediastinal and hilar lymph nodes on imaging, he reported no recent respiratory infections. Even if the patient experienced active pulmonary disease, deterioration into disseminated infection would still be uncommon in an immunocompetent patient.

Although the patient lives in an area highly endemic for *H. capsulatum*, he was not diagnosed with disseminated histoplasmosis for 6 weeks following the onset of symptoms. His travel history to Bolivia complicated the clinical picture, making a virulent tropical disease a diagnostic consideration in a previously healthy host. He underwent an extensive evaluation for infectious diseases before he was evaluated for histoplasmosis. Given that untreated disseminated infection is progressive and can be fatal over 2–12 weeks 1, 2, this case serves as an important reminder that elements of the patient's history can be misleading. Making an accurate and timely diagnosis may involve disregarding salient features of the clinical picture.

Our patient had severe thrombocytopenia that did not respond to IVIG or platelet transfusions (Fig. 5). Although rare, other cases of histoplasmosis complicated by refractory thrombocytopenia have been reported 3, 4, 6. We conducted a search in MEDLINE (from 1950) in order to identify and summarize previously reported cases of histoplasmosis associated with low platelet counts. We used the terms “histoplasmosis,” “thrombocytopenia,” or “pancytopenia” as Medical Subject Headings and searched full‐text article bibliographies to retrieve additional relevant articles. We excluded nonhuman cases, pediatric cases, articles not translated into English, and reports among immunocompromised patients (e.g., patients with HIV). The 13 articles fulfilling inclusion criteria are summarized in Table 1. In total, 45 cases of histoplasmosis and thrombocytopenia have been reported in immunocompetent adults since 1950, including 21 from Vanderbilt University Medical Center 5, 10 from the Christian Medical College in India 7, four from the National Institutes of Health Clinical Center 8, and 10 reports of individual cases 3, 4, 6, 9, 10, 11, 12, 13, 14, 15. Among these reports, demographics and case details were provided for 14 patients; ages ranged from 20 to 77 years (mean of 52), 11 patients (79%) were male, nine (64%) had splenomegaly, and four (29%) died from active histoplasmosis.

**Table 1:** Reported cases of disseminated histoplasmosis and thrombocytopenia in immunocompetent adults

References	Publication	Article summary
Smith and Utz 8	*Annals of Internal Medicine*	Among 21 adults with disseminated histoplasmosis, eight (38%) had thrombocytopenia. Four cases were among immunocompetent adults
Armitage and Sheets 3	*Journal of the American Medical Association*	A 41‐year‐old woman who recovered from histoplasmosis but had persistent thrombocytopenia requiring splenectomy
Goodwin et al. 5	*Medicine (Baltimore)*	Among 84 cases of histoplasmosis reported over 45 years, 21 cases were immunocompetent adults. Profound thrombocytopenia (<5 k/cumm) occurred in 21% of patients with severe histoplasmosis, and in 0 patients with mild or moderate disease
Kucera and Davis 6	*American Journal of Clinical Pathology*	A 20‐year‐old woman with thrombocytopenia and IgG elevation in primary pulmonary histoplasmosis
Hankey and Gulland 9	*Australia New Zealand Journal of Medicine*	A 45‐year‐old Burmese man with disseminated histoplasmosis and thrombocytopenia in Western Australia
Harten et al. 10	*Clinical Investigation*	A 24‐year‐old woman with disseminated histoplasmosis in an immunocompetent European woman
Singh et al. 15	*Postgraduate Medical Journal*	A 50‐year‐old male farmer with disseminated histoplasmosis and pancytopenia who developed bleeding symptoms and expired
Valdivia‐Arenas and Sood Namita 11	*Chest*	A 77‐year‐old farmer from Ohio with disseminated histoplasmosis, severe sepsis, and thrombocytopenia
Deodhar et al. 7	*National Medical Journal of India*	Among 61 cases of histoplasmosis in India, 10 cases were immunocompetent adults with pancytopenia
Subbalaxmi et al. 4	*Medical Mycology Case Reports*	A 56‐year‐old male farmer from southern India with disseminated histoplasmosis, refractory thrombocytopenia, and bleeding manifestations
Kashif et al. 12	*Case Reports Critical Care*	A 34‐year‐old man with sickle‐cell disease who developed *Histoplasma duboisii* infection and did not respond to amphotericin B. He progressed to develop hemophagocytic lymphocytosis, disseminated intravascular coagulation, and cardiorespiratory arrest
Gajendra et al. 14	*Turkish Journal of Haematology*	A 44‐year‐old man with histoplasmosis, pancytopenia, and bilateral adrenal masses
Elbadawi et al. 17	*ID Cases*	A 65‐year‐old man with pulmonary sarcoidosis (not taking corticosteroids) who developed disseminated histoplasmosis with anemia and thrombocytopenia

Three reported cases of thrombocytopenia were refractory to initial treatment 3, 4, 6. Armitage and Sheets 3 describe the case of a 41‐year‐old woman successfully treated for disseminated histoplasmosis who developed thrombocytopenia (platelet count 6 k/cumm) after a month of amphotericin B therapy. A trial dose of prednisone initially improved platelet counts to 100 k/cumm, but steroids could not be weaned and the patient required splenectomy. Kucera and Davis report the case of a 20‐year‐old woman with pulmonary histoplasmosis, thrombocytopenia (platelet count: 5 k/cumm on admission) refractory to platelet transfusions, and an elevated platelet antibody level 6. Following treatment for histoplasmosis, the platelet counts improved without specific therapy and platelet antibodies decreased to normal levels. Subbalaxmi et al. describe the case of a 56‐year‐old man from India with thrombocytopenia (platelet count: 10 k/cumm) and bleeding manifestations. The platelet count continued to drop despite transfusions on alternate days and improved beginning on day 5 of treatment. In our case, the patient's platelet counts were refractory to treatment with both IVIG and platelet transfusions. The platelet count gradually increased until it normalized 2 months after initiation of antifungal therapy.

Several mechanisms have been postulated to contribute to the thrombocytopenia resulting from a histoplasmosis infection. First, with severe infection, *H. capsulatum* can infiltrate the bone marrow and lead to thrombocytopenia, along with anemia and leukopenia, due to impaired production 8. Second, in human plasma, *H. capsulatum* can form a complex with IgG and fibrinogen and promote platelet aggregation and serotonin release 16. Third, histoplasmosis may rarely induce immune thrombocytopenic purpura (ITP), as evidenced by the immunocompetent patient with the delayed severe thrombocytopenia that responded to splenectomy 3. Finally, with disseminated infection, abnormal activation of the coagulation system, as in disseminated intravascular coagulation, may lead to consumption of platelets. In our patient, as well as two other reported cases 4, 6, thrombocytopenia improved dramatically following successful treatment of histoplasmosis. Although our patient had evidence of *H. capsulatum* in the bone marrow, megakaryocytes were present, suggesting that infiltration of the bone marrow alone was likely not a sufficient mechanism to cause such severe thrombocytopenia. Consumption of platelets in an overfunctional immune system and diffuse inflammatory state such as in this case is possible. Our patient had a platelet‐associated antibody screening of 70%, and while this test has low specificity, it implies an autoimmune mechanism for his severe thrombocytopenia. There was a rapid destruction of the transfused platelets as early as 2 hours posttransfusion, which would also suggest an immune mechanism (Fig. 6). The autoimmune response did not resolve until the eradication of histoplasmosis, which suggests that the infection was either directly or indirectly involved in generating this response.

In summary, disseminated histoplasmosis among immunocompetent patients is rare, but may be associated with clinically significant refractory thrombocytopenia. *H. capsulatum* can cause thrombocytopenia by infiltrating bone marrow and affecting platelet production, promoting platelet aggregation, or inducing ITP. Among reported cases of severe thrombocytopenia associated with histoplasmosis, platelet counts often return to normal levels following antifungal therapy. Therefore, the most important management of this refractory thrombocytopenia is the recognition and treatment of histoplasmosis infection.

## Authorship

IK: collected data, wrote original draft, and performed literature review. LV: wrote original draft and performed literature review. MG: contributed to review and editing, validation of infectious disease data, and supervision. MC: contributed to review and editing, preparation of pathology slides, and validation of pathology data. JS and ZF: contributed to review and editing. RM: contributed to review and editing, validation of hematology data, and supervision and provided funding.

## Conflict of Interest

None declared.
